# **TIPS plus sequential systemic therapy of advanced HCC patients with tumour thrombus-related symptomatic portal hypertension**

**DOI:** 10.1007/s00330-022-08705-7

**Published:** 2022-04-20

**Authors:** Zhenkang Qiu, Guobao Wang, Huzheng Yan, Han Qi, Mengxuan Zuo, Guisong Wang, Weiwei Jiang, Zixiong Chen, Jingbing Xue, Ligong Lu, Fujun Zhang, Fei Gao

**Affiliations:** 1 Department of Minimally Invasive & Interventional Radiology, Sun Yat-sen University Cancer Center and Sun Yat-sen University State Key Laboratory of Oncology in South China, and Collaborative Innovation Center for Cancer Medicine, 651 Dongfeng East Road, Guangdong Province Guangzhou, 510060 China; 2 Department of Endoscopy, Sun Yat-sen University Cancer Center and Sun Yat-sen University State Key Laboratory of Oncology in South China, and Collaborative Innovation Center for Cancer Medicine, Guangzhou, Guangdong China; 3 Department of Interventional Radiology, The Third affiliated hospital of Sun Yat-sen University, Guangzhou, China; 4 Department of Imaging Sciences, University of Rochester Medical Center, Rochester, NY USA; 5 Department of Radiology, Zhuhai City People’s Hospital, Zhuhai, Guangdong China

**Keywords:** TIPS, Hepatocellular carcinoma, Portal hypertension

## Abstract

**Objectives:**

Portal vein tumour thrombus (PVTT)–related symptomatic portal hypertension (SPH) leads to a poor prognosis in hepatocellular carcinoma (HCC) patients. A transjugular intrahepatic portosystemic shunt (TIPS) can effectively relieve SPH but its effect remains unclear in PVTT-related SPH. This study aimed to evaluate the clinical value of the TIPS procedure combined with sequential systemic therapy in advanced HCC patients with PVTT-related SPH.

**Methods:**

After 1:1 propensity score matching (PSM), this retrospective study analysed 42 patients who underwent TIPS placement plus sequential systemic therapy (group A) and 42 patients who received only symptomatic and supportive treatment (group B). The evaluated outcomes were overall survival (OS) and SPH control rate. Cox proportional hazards regression analysis was used to compare OS in the two groups.

**Results:**

In group A, the technical success rate of the TIPS procedure was 95.2%, and no severe complications occurred. The rebleeding rates in group A and group B were 5.0% and 73.7%, respectively (*p* < 0.001), and the ascites control rates were 92.0% and 28.0%, respectively (*p* < 0.001). The median OS of group A was significantly better than that of group B (9.6 [95% CI: 7.1, 12.0] *vs.* 4.9 [95% CI: 3.9, 5.8], months, *p* < 0.001). Multivariable analysis showed that TIPS plus sequential systemic therapy (hazard ratio [HR] = 5.799; 95% CI: 3.177, 10.585; *p* < 0.001) was an independent prognostic factor related to OS. Additionally, PVTT degree (I+II) (*p* = 0.008), AFP ≤ 400 ng/ml (*p* = 0.003), and Child–Pugh class A (*p* = 0.046) were significant predictors of OS.

**Conclusion:**

TIPS plus sequential systemic therapy is safe and feasible for treating advanced HCC with tumour thrombus-related SPH.

**Key Points:**

• *Portal vein tumour thrombus (PVTT) is common in advanced hepatocellular carcinoma (HCC) and transforms compensated portal hypertension into symptomatic portal hypertension (SPH).*

• *HCC patients with PVTT-related SPH have a very poor prognosis, and there are no effective treatments recommended by the guidelines.*

• *Therefore, a treatment strategy that utilises a transjugular intrahepatic portosystemic shunt (TIPS) to manage SPH combined with sequential systemic therapy in advanced HCC patients is explored in this study for its feasibility and clinical value. This research can fill the gap in current research data to provide clinically meaningful treatment options.*

**Supplementary Information:**

The online version contains supplementary material available at 10.1007/s00330-022-08705-7.

## Introduction

Hepatocellular carcinoma (HCC) is one of the most common malignancies often connected to liver cirrhosis and portal hypertension [1–3]. It is the predominant cause of cancer-related deaths globally because most patients are diagnosed at an advanced stage with portal vein invasion or portal vein tumour thrombosis (PVTT) [4, 5]. The flow of the portal vein can be blocked by PVTT, thereby aggravating the condition of portal hypertension. PVTT is significantly associated with poor prognosis, leading to symptomatic portal hypertension (SPH) complications such as variceal bleeding, refractory ascites or hydrothorax, and diarrhoea [6, 7]. The median survival time of these patients is 2.7 months without aggressive intervention [8, 9]. New treatment modalities, such as lenvatinib and immune checkpoint inhibitors, have effectively improved the OS of HCC patients with PVTT [10]. However, SPH has led to a more conservative treatment strategy for these patients. It is thus essential to assess the clinical symptoms of portal hypertension to facilitate the development of antitumour treatment regimens.

The transjugular intrahepatic portosystemic shunt (TIPS) is an effective treatment to relieve the complications of portal hypertension in patients with cirrhosis. Various studies have also promoted technical advancements and expanded the indications for complications of portal hypertension, such as early TIPS and non-tumoural portal vein thrombosis [11, 12]. Additionally, previous studies have indicated that TIPS placement could be practical for the transitional treatment of portal hypertension in HCC patients with PVTT. Thus, TIPS placement is envisioned to effectively relieve patients’ symptoms and create the basis for further treatment [13–16]. However, the standard method for treating HCC with PVTT and SPH is still unclear. This study retrospectively assessed the outcomes of HCC patients with PVTT who received TIPS combined with sequential systemic therapy, including its feasibility and safety.

## Materials and methods

### Patients

**Fig. 1:**
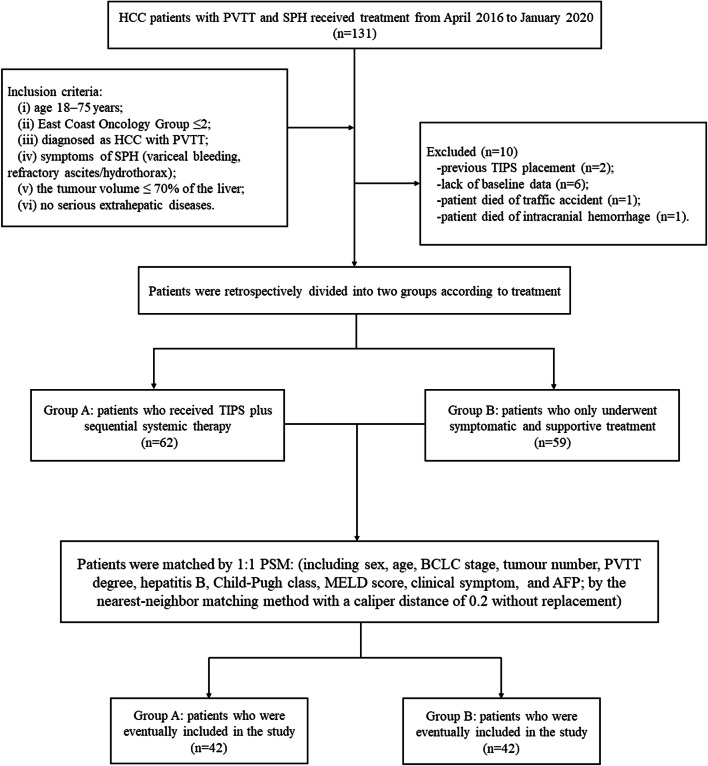
Patient flow diagram. The definition of “no serious extrahepatic diseases” is that patients had no lung metastasis or bone metastasis of HCC, no congestive heart failure, no multiple hepatic cysts, no uncontrolled systemic infection or sepsis, no unrelieved biliary obstruction, no severe pulmonary hypertension, no moderate pulmonary hypertension, and no severe coagulopathy. HCC, hepatocellular carcinoma; PVTT, portal vein tumour thrombus; TIPS, transjugular intrahepatic portosystemic shunt; PSM, propensity score matching; BCLC, Barcelona Clinic Liver Cancer; MELD, Model for End-Stage Liver Disease

This retrospective study was performed in accordance with the Declaration of Helsinki of the World Medical Association. It was approved by the Institutional Review Board, and the requirement for informed consent from patients was waived because of its low risk. All patients provided written informed consent for treatment. Patients were considered for inclusion if they were 18–75 years old; had an East Coast Oncology Group score ≤ 2; met the diagnostic criteria for HCC, PVTT, or SPH; had a tumour volume of less than 70% of the liver volume; and had no lung metastasis or bone metastasis of HCC, no congestive heart failure, no multiple hepatic cysts, no uncontrolled systemic infection or sepsis, no unrelieved biliary obstruction, no severe pulmonary hypertension, no moderate pulmonary hypertension, and no severe coagulopathy. Excluded patients had previous TIPS placement, lacked baseline data, and died of non-tumour-related causes. From April 2016 to January 2020, 131 HCC patients with PVTT and SPH were treated at the interventional radiology centre. After 1:1 propensity score matching (PSM), 42 patients who received TIPS plus sequential systemic therapy (group A), and 42 patients who received only symptomatic and supportive treatment (group B) were eventually included in this study (Fig. 1).

### Diagnosis

The diagnostic criteria of HCC were based on the European Association for the Study of the Liver Clinical Practice Guidelines: Management of Hepatocellular Carcinoma and the American Association for the Study of Liver Diseases guidelines for the treatment of HCC [13, 17]. Computed tomography (CT) or magnetic resonance imaging (MRI) was completed to define the degree of PVTT, and colour Doppler ultrasonography (CDUS) was employed to detect changes in portal flow [12]. The degree of PVTT was based on the Yerdel classification using the following four types: degree I, a thrombus occluding < 50% of the portal vein, with or without minimal obstruction of the superior mesenteric vein (SMV); degree II, a > 50% occlusion of the portal vein, including total occlusions, with or without the minimal extension into the SMV; degree III, complete thrombosis of both the portal vein and the proximal SMV, with the distal SMV remaining open; and degree IV, complete thrombosis of the portal vein and the proximal and distal SMV [18].

The definition of refractory ascites or hydrothorax was based on the following features: (i) unresponsiveness to a limited sodium diet and intensive diuretic therapy; (ii) diuretic intolerance; and (iii) rapid recurrence of ascites or hydrothorax after therapeutic puncture [19]. The efficacy evaluation of TIPS placement for ascites or hydrothorax was based on the following criteria: (i) complete remission, complete disappearance of ascites; (ii) partial remission (ascites exists but does not need to be punctured); and (iii) absent remission (the presence of severe ascites requires repeated puncture) [20].

### TIPS procedure

**Fig. 2:**
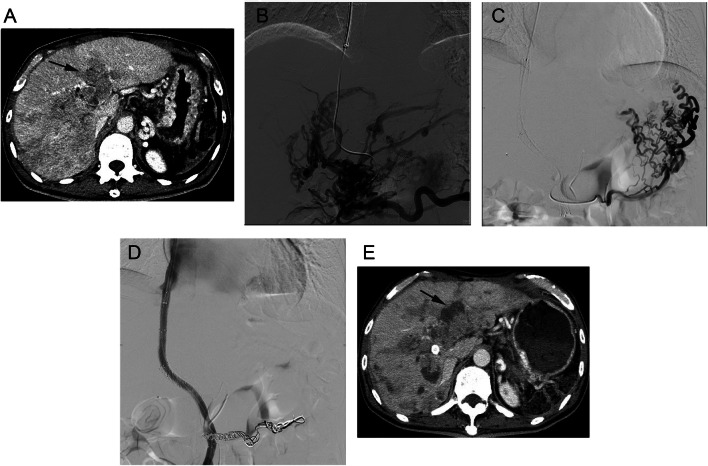
A 55-year-old male patient with hepatocellular carcinoma (HCC) and tumour thrombus-related symptomatic portal hypertension underwent a transjugular intrahepatic portosystemic shunt (TIPS) procedure for severe variceal bleeding and received sequential systemic therapy with lenvatinib (8 mg/qd). **A** Contrast-enhanced computed tomography showed advanced HCC with extensive portal vein tumour thrombus (PVTT) before treatment (black arrowhead). **B** Superior mesenteric venography showed the disappearance of the portal vein and branches that were replaced by disordered collateral veins. The portal venous pressure gradient (PPG) was 28.2 mmHg before TIPS creation. **C** Angiography showed gastroesophageal varices (GOV2). **D** After TIPS creation, angiography showed that the stent was smooth, and the collateral circulation veins were significantly reduced. The portal venous PPG was 11.5 mmHg after TIPS creation. The gastroesophageal varices were embolised with coils, and the varices disappeared on angiography. **E** After 6 months of sequential systemic therapy, the viable lesions of the tumour and PVTT were significantly decreased (black arrowhead), and the TIPS patency was satisfactory. The overall survival period after TIPS placement was 10.8 months without variceal rebleeding

After the internal jugular vein was catheterised with the 10-F sheath, the hepatic vein was selected for angiography. The radiologist used a needle (RUPS-100, COOK) to puncture the portal vein branch to perform portal vein angiography. Then, a wire (HiWire Hydrophilic Wire Guide, COOK) was inserted into the SMV, and balloon angioplasty was performed. Finally, covered stents (VIATORR, Gore & Associates or Fluency Plus, Becton, Dickinson and Company) were placed to cover the narrow segment caused by tumour thrombosis. To ensure adequate blood flow in the distal portal vein, bare stents (LUMINEXX, Becton, Dickinson and Company) were used to recanalise the occlusive thrombus. The portal venous pressure gradient (PPG) was measured before and after shunt creation. The postoperative PPG target was below 12 mmHg. Technical success was defined as the successful creation of a shunt between the hepatic vein and the portal vein via internal jugular vein access. All patients received anticoagulant therapy, rivaroxaban (10–20 mg/day, Bayer Schering Pharma AG), after the procedure for 3–6 months. A representative case is shown in Fig. 2.

### Sequential systemic therapy

**Fig. 3:**
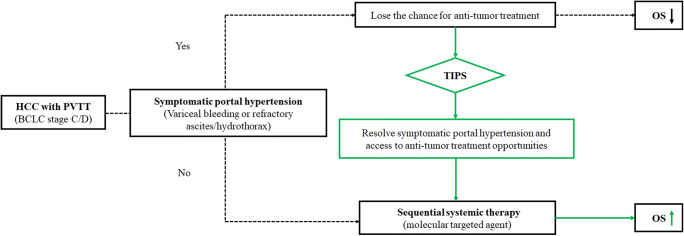
Treatment strategy for advanced hepatocellular carcinoma (HCC) with portal vein tumour thrombus (PVTT)–related symptomatic portal hypertension to improve survival. A transjugular intrahepatic portosystemic shunt (TIPS) is used to resolve portal hypertension complications, including variceal bleeding, refractory ascites or hydrothorax, and access to antitumour treatment opportunities (molecular targeted agents)

All patients in group A received sequential systemic therapy with molecular targeted agents after the TIPS procedure when the symptoms of portal hypertension were controlled. Patients received a daily oral dose of sorafenib (400 mg/bid, Bayer) or lenvatinib (8–12 mg/qd, Eisai Co Ltd). Patients received regorafenib (120–160 mg/qd during weeks 1–3 of each 4-week cycle, Bayer) if they were nonresponders or tolerated sorafenib or lenvatinib. The treatment strategy is shown in Fig. 3.

### Symptomatic and supportive treatment

The patients in group B received only symptomatic and supportive treatment because of the rejection of the TIPS and molecular targeted agent treatment. For variceal bleeding, combined treatment with vasoactive drugs, prophylactic antibiotics, and endoscopic techniques was used. For refractory ascites, large-volume paracentesis administered with albumin and diuretics was used.

### Follow-up

Patients were followed up with laboratory tests (such as blood count, liver function, and coagulation function evaluations) every 2 weeks and alpha-fetoprotein (AFP) tests every month after TIPS placement. Abdominal CT/MRI and CDUS were performed every 2 months after TIPS placement. Chest CT was performed every 2 months. The endpoint of follow-up was death or August 2, 2021. The overall survival (OS) of group A was calculated from the TIPS procedure to death, and the OS of group B was calculated from the symptomatic treatment of SPH to death. Shunt occlusion was considered as the following situations: (i) recurrent variceal bleeding; (ii) recurrent ascites or aggravation; or (iii) blood shunt-flow disappearing or maximum shunt-flow less than 50 cm/s in CDUS [12].

### Statistical analysis

Continuous variables were compared using a *t test*. Pearson *χ*^2^ was used to compare the qualitative data. The Kaplan–Meier method was used to analyse OS, and the log-rank test (Mantel–Cox) was performed to compare OS among different groups. Univariable and multivariable Cox regression analyses were used to identify prognostic factors for OS. Hazard ratio (HR) and their 95% confidence interval (CI) were calculated with Cox regression. To reduce the influence of potential confounding factors on selection bias in this study, the baseline patient data of the two groups (Supplemental Table 1) were matched with 1:1 PSM. Sex, age, hepatitis, Barcelona Clinic Liver Cancer (BCLC) stage, tumour number, PVTT degree, Child–Pugh class, Model for End-Stage Liver Disease (MELD) score, AFP, and clinical symptoms were included in the PSM model (Supplemental Figure 1). The PSM was realised by the nearest-neighbour matching method with a calliper distance of 0.2 without replacement. SPSS Statistics 26.0 (IBM) and R software package 3.5.0 (R Foundation for Statistical Computing) were used for statistical analyses. Differences with a *p* value ≤ 0.05 were considered statistically significant, and all statistical tests were two-sided.

## Results

### Patient characteristics

After PSM, among all 84 HCC patients with PVTT and SPH, 77 were males and 7 were females; the average age was 53.4 years (range, 30–75 years). In total, 77 (91.7%) patients were in BCLC stage C, and 80 (95.2%) patients had hepatitis B virus infection. According to the Child–Pugh class standard, there were 26 (31.0%), 51 (60.7%), and 7 (8.3%) patients with Child–Pugh classes A, B, and C, respectively. The average Child–Pugh score was 7.4 (range, 5–12). Stratified by the PVTT degree, there were 16 (19.0%), 30 (35.7%), 29 (34.5%), and 9 (10.7%) patients with PVTT degrees I, II, III, and IV, respectively. The symptoms of SPH included variceal bleeding (34, 40.5%), refractory ascites and/or hydrothorax (45, 53.6%), and variceal bleeding combined with refractory ascites (5, 6.0%). There was no significant difference in the baseline characteristics of patients in group A and group B. More basic characteristics of the patients after PSM are shown in Table 1.

**Table 1:** Baseline patient characteristics after propensity score matching

Characteristics	All *n* = 84 (%)	Group A *n* = 42 (%)	Group B *n* = 42 (%)	*p* value
Sex				0.693
Male	77 (91.7)	39 (92.9)	38 (90.5)	
Female	7 (8.3)	3 (7.1)	4 (9.5)	
Median age [range], years	53.4 [30.0;75.0]	52.7 [32.0;73.0]	54.0 [30.0;75.0]	0.578
BCLC stage				0.693
C	77 (91.7)	38 (90.5)	39 (92.9)	
D	7 (8.3)	4 (9.5)	3 (7.1)	
Tumour number				0.578
Single	16 (19.0)	9 (21.4)	7 (16.7)	
Multiple	68 (81.0)	33 (78.6)	35 (83.3)	
PVTT degree				0.661
I+II	16 + 30 (54.8)	7 + 15 (52.4)	9 + 15 (57.1)	
III+IV	29 + 9 (45.2)	15 + 5 (47.6)	14 + 4 (42.9)	
Hepatitis B				0.306
Yes	80 (95.2)	41 (97.6)	39 (92.9)	
No	4 (4.8)	1 (2.4)	3 (7.1)	
Child-Pugh class				0.854
A	26 (31.0)	12 (28.6)	14 (33.3)	
B	51 (60.7)	26 (61.9)	25 (59.5)	
C	7 (8.3)	4 (9.5)	3 (7.1)	
Child-Pugh score, [range]	7.4 [5;12]	7.6 [5;12]	7.2 [5;11]	0.262
MELD score, [range]	9.3 [4;15]	9.5 [4;15]	9.1 [6;14]	0.427
MELD score				0.212
≤ 11	72 (85.7)	34 (81.0)	38 (90.5)	
> 11	12 (14.3)	8 (19.0)	4 (9.5)	
Clinical symptom				0.895
Variceal bleeding	34 (40.5)	17 (40.5)	17 (40.5)	
Refractory ascites/hydrothorax	45 (53.6)	22 (52.4)	23 (54.8)	
Variceal bleeding+refractory ascites	5 (6.0)	3 (7.1)	2 (4.8)	
AFP (ng/ml)				0.821
≤ 400	31 (36.9)	16 (38.1)	15 (35.7)	
> 400	53 (63.1)	26 (61.9)	27 (64.3)	

Notes: Unless otherwise indicated, data are the number of patients, with percentages in parentheses; Group A, transjugular intrahepatic portosystemic shunt (TIPS) plus sequential systemic therapy; Group B, only symptomatic and supportive treatment. *p* value ≤ 0.05 was considered to indicate statistical significance

Abbreviations: *BCLC* Barcelona Clinic Liver Cancer, *PVTT*, portal vein tumour thrombus, *MELD* Model for End-Stage Liver Disease, *AFP* alpha-fetoprotein

### Survival analysis

**Fig. 4:**
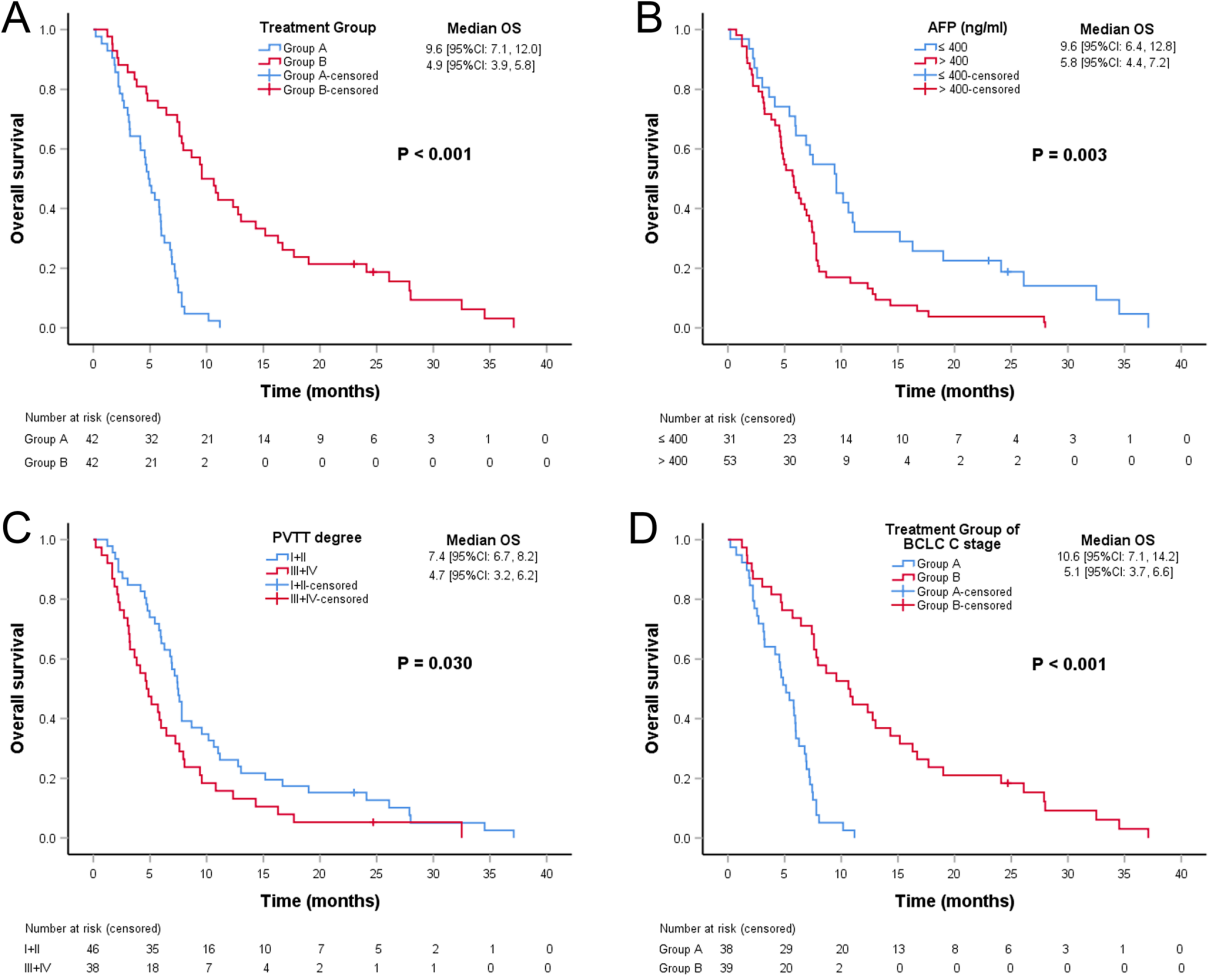
Kaplan–Meier analysis of overall survival (OS). **A** Comparison of the OS of group A (transjugular intrahepatic portosystemic shunt [TIPS] plus sequential systemic therapy) and group B (only symptomatic and supportive treatment). **B** Comparison of the OS of patients with AFP ≤ 400 ng/ml and AFP > 400 ng/ml. **C** Comparison of the OS of patients with PVTT degree (I+II) and PVTT degree (III+IV). **D** Comparison of the OS of group A and group B in patients with BCLC stage C

During follow-up, 40 of 42 (95.2%) patients in group A died of advanced HCC, and 42 of 42 (100%) patients in group B died of advanced HCC or complications of SPH. The median OS of group A was significantly better than that of group B (9.6 [95% CI: 7.1, 12.0] *vs.* 4.9 [95% CI: 3.9, 5.8], months, *p* < 0.001) (Fig. 4, A). Multivariable analysis showed that TIPS plus sequential systemic therapy (HR= 5.799; 95% CI: 3.177, 10.585; *p* < 0.001) was an independent prognostic factor related to OS. Additionally, PVTT degree (I+II) (HR = 0.536; 95% CI: 0.338, 0.852; *p* = 0.008), AFP ≤ 400 ng/ml (HR=0.575; 95% CI: 0.350, 0.947; *p* = 0.003), and Child–Pugh Class A (HR = 0.588; 95% CI: 0.350, 0.990; *p* = 0.046) were significant predictors of OS (Table 2). The median OS of patients with AFP ≤ 400 ng/ml was significantly better than that of patients with AFP > 400 ng/ml (9.6 [95% CI: 6.4, 12.8] *vs.* 5.8 [95% CI: 4.4, 7.2], months, *p* = 0.003) (Fig. 4, B). The median OS of patients with PVTT degree (I+II) was significantly better than that of patients with PVTT degree (III+IV) (7.4 [95% CI: 6.7, 8.2] *vs.* 4.7 [95% CI: 3.2, 6.2], months, *p* = 0.030) (Fig. 4, C). Especially for patients with BCLC stage C, the median OS of group A was significantly better than that of group B (10.6 [95% CI: 7.1, 14.2] *vs.* 5.1 [95% CI: 3.7, 6.6], months, *p* < 0.001) (Fig. 4, D).

**Table 2:** Univariate and multivariate analysis of factors associated with overall survival

**Variables**	**Univariate analysis**			**Multivariate analysis**		
	**HR**	**95% CI**	*p* value	**HR**	**95% CI**	*p* value
Age ≤ 60 years	1.193	0.691, 2.060	0.527			
Male	0.541	0.229, 1.281	0.163			
Single tumour	0.667	0.357, 1.246	0.204			
PVTT degree (I+II)	0.624	0.379, 1.025	0.063	0.536	0.338, 0.852	0.008^*^
Hepatitis B	0.463	0.153, 1.399	0.172			
AFP ≤ 400 ng/ml	0.559	0.319, 0.982	0.043	0.575	0.350, 0.947	0.003^*^
Variceal bleeding	1.075	0.553, 2.088	0.832			
Child-Pugh class A	0.658	0.314, 1.377	0.267	0.588	0.350, 0.990	0.046*
BCLC C stage	0.632	0.249, 1.606	0.632			
MELD score ≤ 11	1.609	0.754, 3.435	0.219	1.778	0.912, 3.468	0.091
Treatment group B	5.994	3.222, 11.153	< 0.001	5.799	3.177, 10.585	< 0.001^*^

Notes: Treatment group B, patients without TIPS plus sequential systemic therapy, only symptomatic and supportive treatment; ^*^ *p* value ≤ 0.05 was considered to indicate statistical significance

Abbreviations: *HR* hazard ratios, *CI* confidence interval, *PVTT* portal vein tumour thrombus, *AFP* alpha-fetoprotein, *BCLC* Barcelona Clinic Liver Cancer, *MELD* Model for End-Stage Liver Disease

### Technical success of TIPS

For group A, the TIPS procedure was implemented owing to variceal bleeding (17, 40.5%), refractory ascites and/or hydrothorax (22, 52.4%), and 3 (7.1%) patients had variceal bleeding combined with ascites. TIPS insertion was performed only via the internal jugular vein approach in 40 patients. Technical success was achieved in 40 of 42 (95.2%) sessions, and the other two patients underwent TIPS placement combined with a transhepatic approach because of poor indirect portography or lack of imaging of the portal vein system. In six cases, bare stents (LUMINEXX, Becton, Dickinson and Company) were used to recanalise the blocked portal vein. All bare stents had a diameter of 8 mm, with lengths of 80 mm and 100 mm. For the dilated gastric or oesophageal varices, embolisation with coils was performed via the coronary ventricular vein. After TIPS creation, the mean PPG was reduced from 25.2 ± 6.29 mmHg to 10.8 ± 6.59 mmHg (Table 3).

**Table 3:** Characteristics and TIPS-related complications in Group A

Characteristics	*n*	%
Transhepatic approach		
No/yes	40/2	95.2/4.8
Arterioportal fistula		
No/yes	40/2	95.2/4.8
TIPS-revision		
No/yes	39/3	92.9/7.1
Coronary vein embolisation		
No/yes	11/31	26.2/73.8
PPG (mmHg) ^†^		
Before TIPS	25.2 ± 6.29	
After TIPS	10.8 ± 6.59	
Reducing	14.3 ± 4.31	
Systemic therapy		
Sorafenib/lenvatinib	23/19	54.8/45.2
Regorafenib	5	11.9
TIPS-related complications		
Intraperitoneal haemorrhage	1	2.4
Bile duct injury	1	2.4
Hepatic encephalopathy		
Mild/moderate/severe	2/2/1	4.8/4.8/2.4
Abnormal liver function after TIPS creation	2	4.8

Notes: † mean ± standard deviation; Unless otherwise indicated, data are the number of patients, with percentages in parentheses; Group A, TIPS plus sequential systemic therapy

Abbreviations: *TIPS* transjugular intrahepatic portosystemic shunt, *PPG* portal venous pressure gradient

### TIPS-related complications and adverse events of treatment with molecular targeted agents

TIPS-related complications in group A are presented in Table 3. One patient had an intraperitoneal haemorrhage after TIPS creation, probably because of injury to the hepatic capsule. One patient had a duodenal papilla haemorrhage visible through endoscopy, caused by bile duct injury due to puncture. In two patients, alanine aminotransferase and aspartate aminotransferase values significantly increased. After 3–5 days of hospitalisation, the alanine aminotransferase and aspartate aminotransferase levels decreased. All patients in group A had no symptoms of hepatic encephalopathy (HE) before the TIPS procedure. Four (9.5%) patients developed mild or moderate HE after TIPS placement during the follow-up period, and these symptoms improved through internal medical treatment. Another (2.4%) patient underwent the second procedure with a reducing stent because of severe HE. Shunt occlusion occurred at least once after TIPS creation in three (7.1%) patients in group A. These patients received a TIPS revision to recanalise the shunt. Patients received oral rivaroxaban (10–20 mg/day) after TIPS revision.

After variceal bleeding and refractory ascites/hydrothorax control, 23 (54.8%) patients and 19 (45.2%) patients received sorafenib and lenvatinib, respectively. Five of them received regorafenib because of nonresponse or tolerance. The rates of adverse events (≤ grade 3) of treatment with molecular targeted agents were 23.8%, 28.6%, 38.1%, 28.6%, and 9.5%, for hypertension, palmar-plantar erythrodysesthesia, fatigue, decreased appetite, and diarrhoea, respectively. Severe complications, such as tumour rupture, severe intra-abdominal haemorrhage, or death, were not observed (Table 4).

**Table 4:** Treatment adverse events of molecular targeted agents in Group A

Adverse events	*n* (%)			
	Any grade	Grade 1	Grade 2	Grade 3
Hypertension	10 (23.8)	2 (4.8)	6 (14.3)	2 (4.8)
Palmar-plantar erythrodysaesthesia	12 (28.6)	9 (21.4)	2 (4.8)	1 (2.4)
Fatigue	16 (38.1)	8 (19.0)	6 (14.3)	2 (4.8)
Decreased appetite	12 (28.6)	7 (16.7)	4 (9.5)	1 (2.4)
Diarrhoea	4 (9.5)	3 (7.1)	1 (2.4)	0

Notes: Data are the number of patients, with percentages in parentheses; Group A, TIPS plus sequential systemic therapy. Adverse events grade 4 or 5 were not observed

### Symptom control

A total of 95.0% (19/20) of patients in group A with variceal bleeding had cases that were effectively controlled, but 73.7% (14/19) of patients in group B experienced variceal rebleeding. The Pearson *χ*^2^ test showed that there was a significant difference in the variceal bleeding control rate between the two groups (*p* < 0.001). A total of 72.0% (18/25) and 20.0% (5/25) of patients in group A with refractory ascites and/or hydrothorax achieved complete and partial remission, respectively. Only 28.0% (7/25) of patients with refractory ascites and/or hydrothorax achieved partial remission in group B. There was a significant difference in the remission rate of ascites/hydrothorax between the two groups (*p* < 0.001). The results are shown in Table 5.

**Table 5:** Comparison of symptom control rate of two groups

Symptoms	Group A *n* (%)	Group B *n* (%)	*p* value
Variceal bleeding	20	19	< 0.001^*^
Control	19 (95.0%)	5 (26.3%)	
Rebleeding	1 (5.0%)	14 (73.7%)	
Refractory ascites/hydrothorax	25	25	< 0.001^*^
Complete remission	18 (72.0%)	0 (0.0%)	
Partial remission	5 (20.0%)	7 (28.0%)	
No remission	2 (8.0%)	18 (72.0%)	

Notes: Data are the number of patients, with percentages in parentheses; Group A, transjugular intrahepatic portosystemic shunt (TIPS) plus sequential systemic therapy; Group B, only symptomatic and supportive treatment. ^*^ *p* value ≤ 0.05 was considered to indicate statistical significance

## Discussion

In this study, the concept of “tumour thrombus–related portal hypertension” was emphasised, which might be further classified as symptomatic and nonsymptomatic. For nonsymptomatic patients with BCLC stage C, systemic therapy such as sorafenib or lenvatinib is recommended as first-line treatment [21–23]. Recent studies have also recommended molecular targeted drugs combined with immune checkpoint inhibitors [10, 24]. However, for symptomatic patients, the use of systemic therapy is limited by the complication of portal hypertension. In most cases, HCC patients with BCLC stage C/D ultimately die due to portal hypertension complications caused by obstruction by a portal vein thrombus. These complications include serious variceal bleeding, refractory ascites, or hepatic failure, but not extensive metastasis [7, 14].

The guideline or consensus on the intervention of SPH in HCC patients with PVTT remains unclear. Because the TIPS procedure has been developed as a critical, minimally invasive therapy for the treatment of complications of portal hypertension [17, 25] the feasibility and clinical value of TIPS combined with sequential systemic therapy were explored in this study. The median OS of group A was 9.6 months, which was higher than that of group B (4.9 months), and the reported results were obtained without aggressive intervention (2.7 months) [8, 9]. This discrepancy may be attributable to the fact that these patients underwent sequential systemic therapy after TIPS insertion. In the case of variceal bleeding, TIPS placement yields a higher control rate of acute bleeding and rebleeding than endoscopic techniques and conservative medical therapy [26–28]. Additionally, the TIPS procedure has an advantage over large paracentesis for refractory ascites related to portal hypertension [29, 30]. Based on the results of this investigation, PVTT degree (I+II), AFP ≤ 400 ng/ml, and liver function with Child–Pugh class A were significant factors for better OS. Notably, the prognosis of HCC patients was influenced by the tumour burden and the degree of liver dysfunction [31]. For SPH in patients with PVTT, TIPS creation can reduce certain life-threatening complications and provide a chance for sequential systemic therapy, which is likely to prolong survival.

In addition, the technical success rate in this study was 95.2%. Only two patients with severe portal cavernoma showed significant improvement after TIPS placement was performed in combination with the transhepatic approach [32]. The results of this study indicate that the TIPS procedure is safe for HCC patients with PVTT and does not increase the incidence of procedure-related complications. Following current guidelines of TIPS management in the treatment of portal hypertension is also recommended, including indications such as early TIPS insertion for acute variceal bleeding, a rescue TIPS for acute variceal bleeding after medical therapy and endoscopic techniques, secondary prevention variceal bleeding, and refractory ascites or hydrothorax [33, 34]. Furthermore, patients should be better graded and individualised in the risk management of portal hypertension. For dilated gastric or oesophageal varices, coronary ventricular vein embolisation should be performed to reduce rebleeding risk [35]. Based on the results of this research, a therapeutic strategy was given preference for better treatment of patients with stable intrahepatic tumours and severe SPH. Two cases of tumour thrombus–related portal hypertension were caused by arterioportal fistula due to tumour invasion, which is called “dynamic portal hypertension” [36, 37]. The portal vein pressure in such cases is usually very high because of the connection between the hepatic artery branches and the portal vein. Reducing the portal vein pressure before TIPS is essential, although transarterial embolisation is not satisfactory for the fistula.

This study has some limitations. First, only 84 patients were included in this research. A more significant number of cases and longer follow-up are necessary for survival analysis in future studies. Second, the TIPS procedure was restricted in use because it is often challenging when the main portal vein is completely occluded, especially when a portal cavernoma forms. Based on our experience, the TIPS procedure is performed with the recanalisation of the portal vein through a transhepatic approach. Finally, this was a single retrospective study, and advanced prospective studies are needed to verify the above findings.

In conclusion, TIPS plus sequential systemic therapy is safe and feasible for treating tumour thrombus-related SPH in advanced HCC and may be an effective supplement to current advanced HCC treatments.

## Supplementary information

ESM 1 (DOCX 230 kb)

## Abbreviations

AFP: Alpha-fetoprotein
BCLC: Barcelona Clinic Liver Cancer
CDUS: Colour Doppler ultrasonography
CT: Computed tomography
HCC: Hepatocellular carcinoma
HR: Hazard ratio
MELD: Model for End-Stage Liver Disease
MRI: Magnetic resonance imaging
OS: Overall survival
PVTT: Portal vein tumour thrombus
SPH: Symptomatic portal hypertension
TIPS: Transjugular intrahepatic portosystemic shunt

## Declarations

### Guarantor

The scientific guarantors of this publication are Fei Gao and Fujun Zhang.

### Statistics and biometry

No complex statistical methods were necessary for this paper.

### Methodology

• retrospective

• case-control study

• performed at one institution
