# Supplementary material for: TIPS plus sequential systemic therapy of advanced HCC patients with tumour thrombus-related symptomatic portal hypertension
Source: Eur Radiol. 2022 Apr 20;32(10):6777–87. doi: 10.1007/s00330-022-08705-7 (PMC9474440; doi:10.1007/s00330-022-08705-7)
Supplement: Supplementary file 1 — (DOCX 230 kb) [file 330_2022_8705_MOESM1_ESM.docx]

**Supplemental Table 1**

Baseline patient characteristics before propensity score matching

| **Characteristics** | **All**  **n=121 (%)** | **Group A**  **n=62 (%)** | **Group B**  **n=59 (%)** | ***P*-value** |
| --- | --- | --- | --- | --- |
| Sex |  |  |  | 0.388 |
| Male | 110 (90.9) | 55 (88.7) | 55 (93.2) |  |
| Female | 11 (9.1) | 7 (11.3) | 4 (6.8) |  |
| Median age [range], years | 54.6 [30.0;75.0] | 55.9 [32.0;73.0] | 53.1 [30.0;75.0] | 0.161 |
| BCLC stage |  |  |  | 0.689 |
| C | 110 (90.9) | 57 (91.9) | 53 (89.8) |  |
| D | 11 (9.1) | 5 (8.1) | 6 (10.2) |  |
| Tumour number |  |  |  | 0.236 |
| Single | 26 (21.5) | 16 (25.8) | 10 (16.9) |  |
| Multiple | 95 (78.5) | 46 (74.2) | 49 (83.1) |  |
| PVTT degree |  |  |  | 0.435 |
| I+II | 22+50 (59.5) | 12+27 (62.9) | 10+23 (55.9) |  |
| III+IV | 36+13 (40.5) | 16+7 (37.1) | 20+6 (44.1) |  |
| Hepatitis B |  |  |  | 0.264 |
| Yes | 112 (92.6) | 59 (95.2) | 53 (89.8) |  |
| No | 9 (7.4) | 3 (4.8) | 6 (10.2) |  |
| Child-Pugh class |  |  |  | 0.514 |
| A | 34 (28.1) | 15 (24.2) | 19 (32.2) |  |
| B | 76 (62.8) | 42 (67.7) | 34 (57.6) |  |
| C | 11 (9.1) | 5 (8.1) | 6 (10.2) |  |
| Child-Pugh score, [range] | 7.6 [5;13] | 7.7 [5;13] | 7.4 [5;11] | 0.296 |
| MELD score, [range] | 9.3 [4;15] | 9.3 [4;15] | 9.3 [4;15] | 0.903 |
| MELD score |  |  |  | 0.895 |
| ≤ 11 | 102 (84.3) | 52 (83.9) | 50 (84.7) |  |
| > 11 | 19 (15.7) | 10 (16.1) | 9 (15.3) |  |
| Clinical symptom |  |  |  | 0.285 |
| Variceal bleeding | 54 (44.6) | 32 (51.6) | 22 (37.3) |  |
| Refractory ascites/hydrothorax | 58 (47.9) | 26 (41.9) | 32 (54.2) |  |
| Variceal bleeding+refractory ascites | 9 (7.4) | 4 (6.5) | 5 (8.5) |  |
| AFP (ng/ml) |  |  |  | 0.455 |
| ≤ 400 | 43 (35.5) | 24 (38.7) | 19 (32.2) |  |
| > 400 | 78 (64.5) | 38 (61.3) | 40 (48.8) |  |

**Notes:** Unless otherwise indicated, data are the number of patients, with percentages in parentheses; Group A, transjugular intrahepatic portosystemic shunt (TIPS) plus sequential systemic therapy; Group B, only symptomatic and supportive treatment. *P*-value ≤ 0.05 was considered to indicate statistical significance.

**Abbreviations:** BCLC, Barcelona Clinic Liver Cancer; PVTT, portal vein tumour thrombus; MELD, Model for End-Stage Liver Disease; AFP, alpha-fetoprotein.

**Supplemental Figure 1**


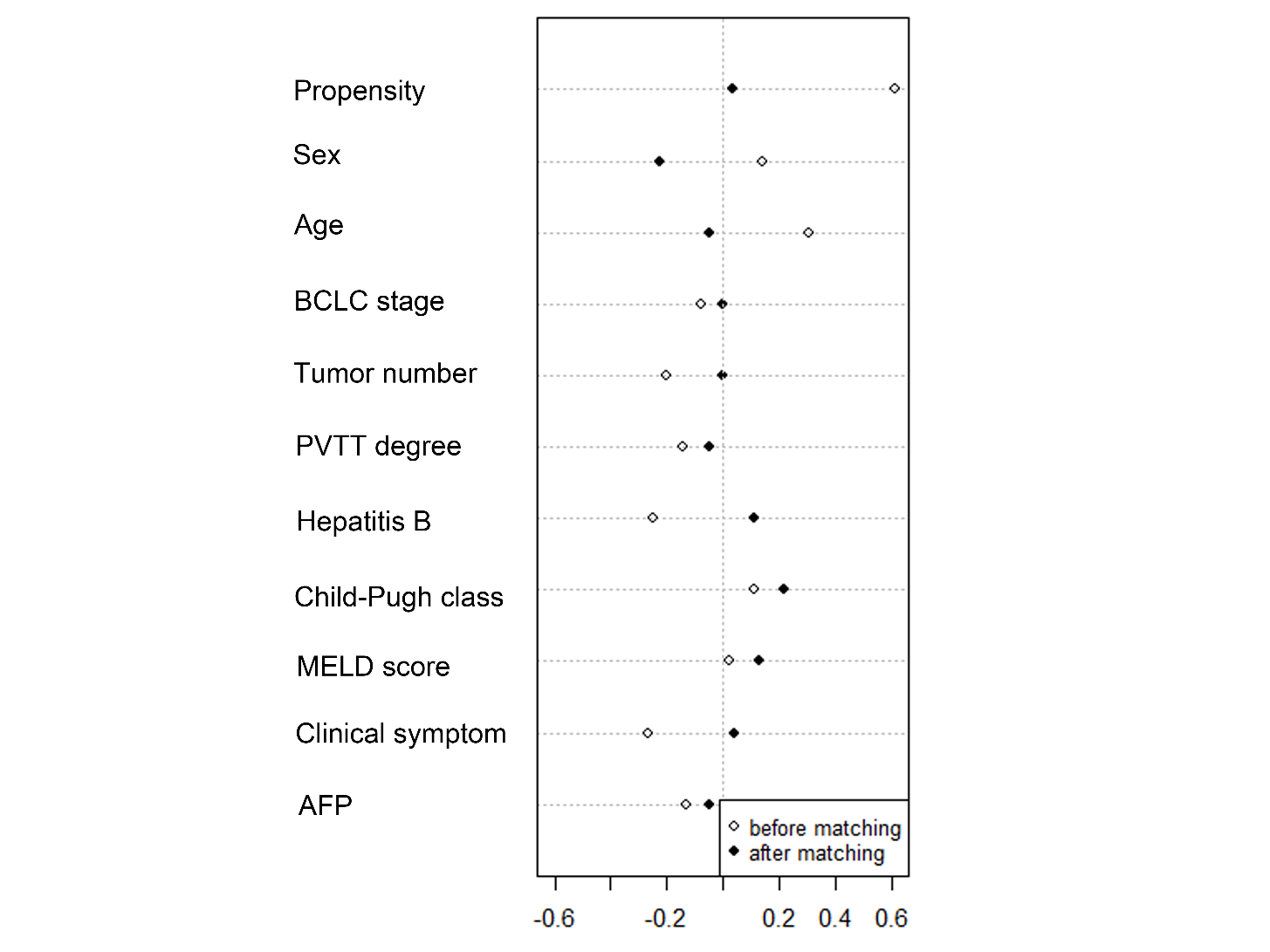


Supplemental Figure 1. R Gragh shows the propensity score of patients’ characteristics before and after matching. Note. Patients were matched by 1:1 propensity score matching (PSM): (including sex, age, BCLC stage, tumour number, PVTT degree, hepatitis B, Child-Pugh class, MELD score, clinical symptom, and AFP; by the nearest-neighbor matching method with a caliper distance of 0.2 without replacement).
